# Mitochondrial DNA damage and subsequent activation of Z-DNA binding protein 1 links oxidative stress to inflammation in epithelial cells

**DOI:** 10.1038/s41598-018-19216-1

**Published:** 2018-01-17

**Authors:** Bartosz Szczesny, Michela Marcatti, Akbar Ahmad, Mauro Montalbano, Attila Brunyánszki, Sofia-Iris Bibli, Andreas Papapetropoulos, Csaba Szabo

**Affiliations:** 1 Department of Anesthesiology, University of Texas Medical Branch, Galveston, TX USA; 2 Center for Biomedical Engineering, University of Texas Medical Branch, Galveston, TX USA; 3 Faculty of Pharmacy, University of Athens, Athens, Greece; 4 Center of Clinical, Experimental Surgery & Translational Research, Biomedical Research Foundation of the Academy of Athens, Athens, Greece

## Abstract

This report identifies mitochondrial DNA (mtDNA) as a target and active mediator that links low-level oxidative stress to inflammatory response in pulmonary epithelial cells. Extrusion of mtDNA into the bronchoalveolar lavage fluid occurs as an early event in mice subjected to cigarette smoke injury, concomitantly with the depletion of mtDNA in the lung tissue. In cultured lung epithelial cells, prolonged, low-level oxidative stress damages the mtDNA, without any detectable damage to the nuclear DNA. In turn, cellular depletion of the mtDNA occurs, together with a transient remodeling of cellular bioenergetics and morphology - all without any detectable impairment in overall cell viability. Damaged mtDNA first enters the cytoplasm, where it binds to Z-DNA binding protein 1 (ZBP1) and triggers inflammation via the TANK-binding kinase 1 /interferon regulatory factor 3 signaling pathway. Fragments of the mtDNA are subsequently released into the extracellular space via exosomes. MtDNA-containing exosomes are capable of inducing an inflammatory response in naïve (non-oxidatively stressed) epithelial cells. *In vivo*, administration of isolated mtDNA into the in lungs of naïve mice induces the production of pro-inflammatory mediators, without histopathologic evidence of tissue injury. We propose that mtDNA-specific damage, and subsequent activation of the ZBP1 pathway, is a mechanism that links prolonged, low-level oxidative stress to autocrine and paracrine inflammation during the early stages of inflammatory lung disease.

## Introduction

The airway epithelium forms the first line of defense to bacteria, viruses, allergens, and air pollutants. Although pulmonary epithelial cells are well-equipped to respond to the environmental insult, chronic exposure to toxicants can lead to inflammatory lung diseases^1,2^. Prolonged, low-level oxidative stress is a key pathogenetic factor in the development of various inflammatory diseases, including chronic obstructive pulmonary disease, asthma and lung cancer^3–6^. Still, the molecular mechanisms linking low-level oxidative stress to inflammation are poorly understood. Mitochondria are multifaceted organelles that are not only key hubs of cellular metabolism and signaling but also important players in response to pathogens and cellular damage^7,8^.

Mitochondrial DNA (mtDNA), liberated from dying (necrotic) cells, belongs to a group of molecules classified as damage-associated molecular pattern (DAMP) molecules. It has been previously shown that mtDNA can induce inflammatory response, particularly in the immune cells^8–10^. MtDNA stimulates polymorphonuclear neutrophils by binding to Toll-like receptor 9 (TLR9) with subsequent activation of the p38 MAP kinase signaling pathway^11^. Moreover, interferon-stimulated gene expression, in response to the activation of the DNA-specific sensor, cGAS, has been linked to mtDNA-induced antiviral immune response^12–14^. During apoptosis, oxidized mtDNA activates nucleotide-binding domain leucine-rich repeat family, pyrine domain containing 3 (NLRP3) inflammasome to produce pro-inflammatory mediators^15,16^.

Although the above-mentioned studies indicate that mtDNA can induce inflammation, these processes were also linked to the death of inflammatory/immune cells, where the cellular content is released into the extracellular space^10^. In contrast, the potential role for mtDNA damage in response to low (non-cytotoxic) levels of oxidative stress is not clear. MtDNA is more sensitive to oxidative damage than nuclear DNA and mtDNA damage takes longer to repair^17–19^. Although persistent accumulation of the unrepaired mtDNA damage can activate apoptotic cell death^20^, the cellular response to the transient mtDNA-specific damage, particularly in parenchymal cells, is largely unknown.

Extracellular, bacterial DNA-induced signaling processes are being intensively investigated in the context bacterial infection and immune responses^21–23^. We hypothesized that mtDNA (which has an evolutionary bacterial origin) may serve as a sentinel molecule to link low-level of oxidative damage to inflammation in parenchymal cells. Using *in vitro* and *in vivo* models, we show that chronic, low level of oxidative stress induces preferential damage to the mtDNA. MtDNA is subsequently released to the cytoplasm and triggers inflammation via the activation of Z-DNA binding protein 1 (ZBP1). This process occurs at non-cytotoxic levels of oxidative stress, and does not require a breakdown of the plasma membrane or a loss of cellular viability. Damaged mtDNA is also actively extruded from cells via exosomes and is capable of inducing inflammation in naïve pulmonary epithelial cells.

## Results

### Mitochondrial DNA-specific damage triggers inflammation

**Figure 1:**
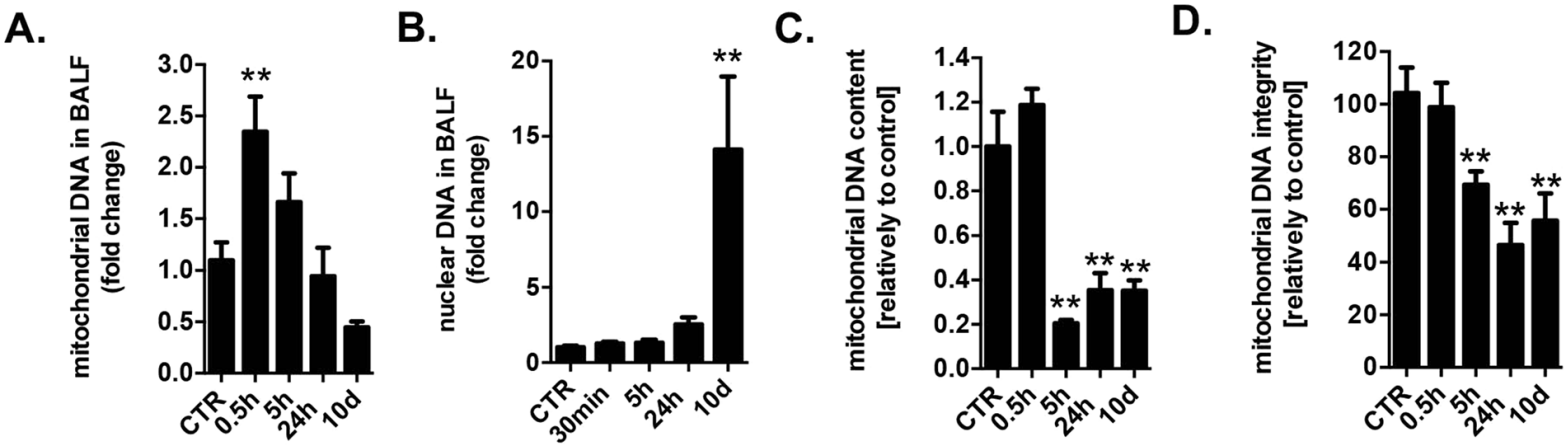
Mitochondrial DNA is released into the bronchoalveolar lavage fluid as an early event in a murine model of cigarette smoke induced lung injury. Early presence of mtDNA (**A**) but not of nuclear DNA (**B**) in BALF of mice exposed to cigarette smoke induced lung injury. MtDNA content is depleted (**C**) and mtDNA integrity is impaired (**D**) in the lung tissue of smoke injured mice. 6–8 animals were used for each experimental end-point. Data represent average ± SEM. **p < 0.01 *vs*. control.

The connection between cigarette smoke injury, oxidative stress and inflammatory diseases is well established. Cigarette smoke induces oxidative stress, which leads to chronic airway inflammation^24,25^. As early as 30 min after cigarette smoke exposure of mice, significant amounts of mtDNA were detected in the bronchoalveolar lavage fluid (BALF) (Fig. 1A). In contrast, nuclear DNA release was not detected until later time points (10 days), most likely due to tissue necrosis (Fig. 1B). Concurrently with mtDNA release, a significant depletion of mtDNA in the lung tissue was measured (Fig. 1C) as well as a decreased integrity of mtDNA (Fig. 1D). In the present model of lung injury, the first detectable sign of lung injury occurs at 3 days^26,27^. Thus, we examined role of mtDNA release as an early contributor to the pathophysiological sequelae of events by analysis of the consequence of the cellular depletion of mtDNA, and the potential role of the extracellular mtDNA in a cultured lung epithelial cell model.

**Figure 2:**
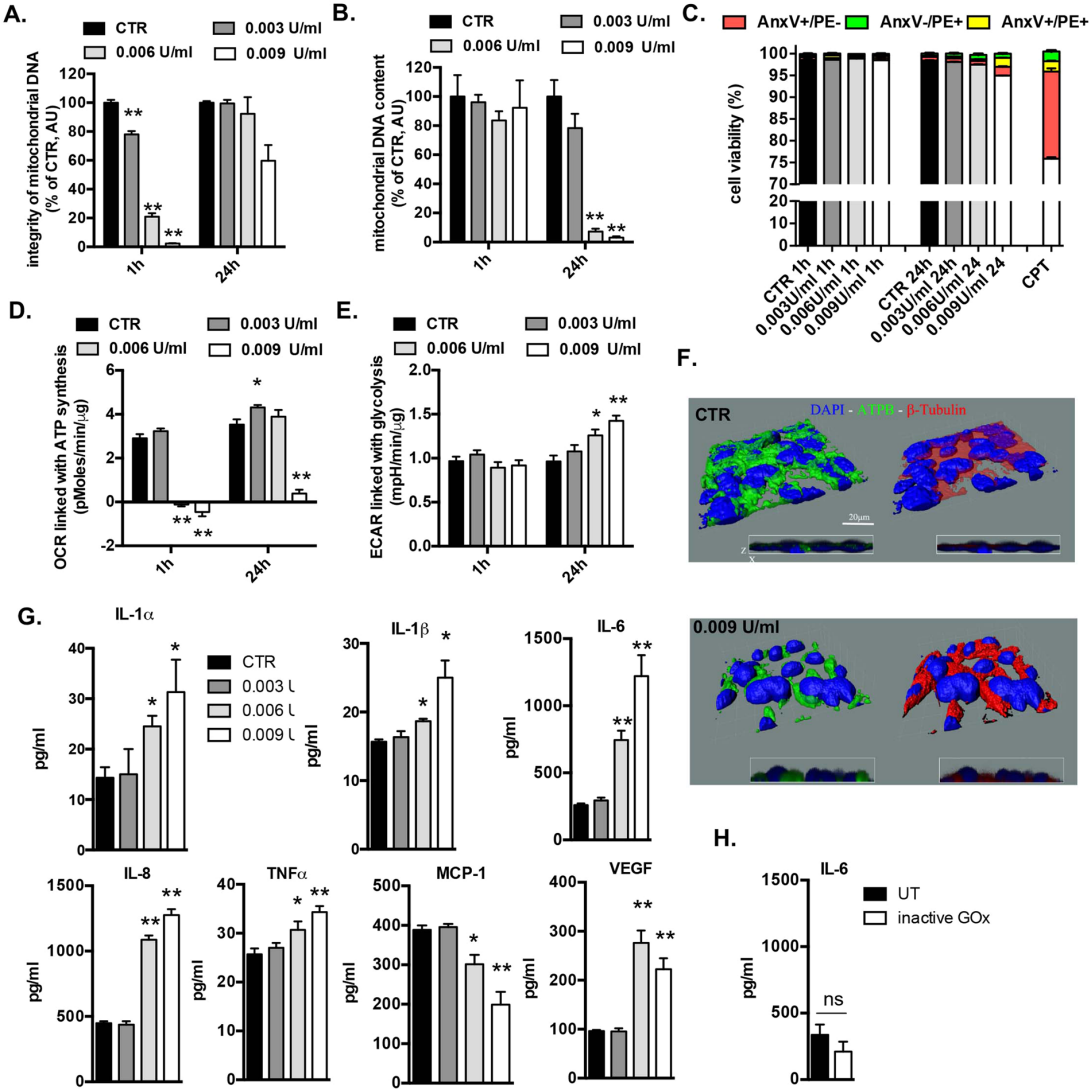
Non-cytotoxic levels of oxidative stress induce mitochondrial DNA depletion, impair cellular bioenergetics and stimulate inflammation. BEAS2B cells were treated with several concentrations of GOx for 1 h and immediately post-challenge as well as 24 h later were measured: (**A**) mitochondrial DNA integrity, (**B**) mitochondrial DNA content, (**C**) apoptotic/necrotic cell death; (**D**) mitochondrial respiration; (**E**) glycolysis. (**F**) 3-D reconstruction of cellular morphology of BEAS2B at 24 h after GOx-treatment was visualized using ATPA-specific antibody (green), β-tubulin (red) and nucleus (DAPI, blue). (**G**) Amount of pro-inflammatory mediators in medium of BEAS2B cells measured at 24 h post GOx-treatment. Data represent average ± SEM of n = 5 biological replicates. (**H**) Lack of enhanced production of IL-6 in BEAS 2B cells stimulated with inactive GOx at 24 h. Representative data of n = 3 independent experiments are shown. *p < 0.05, **p < 0.01 *vs*. control.

To induce prolonged, low-level oxidative stress, lung epithelial cells (BEAS 2B) were incubated with low concentrations of glucose oxidase (GOx), which generates a constant flux of H_2_O_2_ by utilizing glucose present in the culture medium (Fig. S1A). A concentration dependent generation of intracellular oxidative stress by GOx was confirmed using a cell-permeable 2′,7′-dichlorodihydrofluorescein diacetate (H2DCFDA) (Fig. S1B). Next, the degree of DNA damage induced by GOx was measured with long amplicon quantitative PCR-based (LA-qPCR) method which detects mostly DNA breaks^28^. Exposure of the BEAS 2B cells to 0.003, 0.006 or 0.009 U/ml of GOx for 1 h resulted in a concentration-dependent decrease in the mitochondrial but not nuclear DNA integrity (Figs 2A and S1C) but, at this early time point, did not affect mtDNA content (Fig. 2B). We calculated that 0.003, 0.006 or 0.009 U/ml of GOx results in 0.27 ± 0.025, 1.75 ± 0.14 and 4.23 ± 0.84 DNA lesions/10 kb of mtDNA, respectively. While mtDNA damage was subsequently repaired (mtDNA integrity recovered at 24 h post GOx treatment) (Fig. 2A), mtDNA content of the cells was markedly reduced at later time points (Fig. 2B). We did not detect any significant changes in cell viability measured by Annexin V-PE staining (Fig. 2C), plasma membrane integrity (Fig. S1D), mitochondrial volume measured by specific activity of citrate synthase (Fig. S1E), or mitochondrial membrane potential (Fig. S1F) either immediately after GOx treatment nor at 24 h later.

Oxidative stress-induced mtDNA damage was associated with a decrease in mitochondrial respiration (Figs 2D and S1G), with a compensatory increase in glycolysis at 24 h (Figs 2E and S1H). In addition, GOx-treatment resulted in an increased production of mitochondria-derived superoxide as measured at 1 h post-treatment, as evidenced by increased oxygen consumption rate (OCR) linked with proton leak (Fig. S1G) and also confirmed by staining with mitochondrial superoxide specific dye, MitoSOx (Fig. S1I). Although either necrotic nor apoptotic cell death was not measureable (Fig. 2C), we found subtle changes in morphology of oxidatively stressed pulmonary epithelial cells; “cellular shrinkage” and perinuclear accumulation of the mitochondria were noticed at 24 h post GOx-treatment (Figs 2F and S2A,B,C). The majority of measured parameters in response to mild and moderate levels of mtDNA damage (0.003 and 0.006 U/ml of GOx, respectively) were fully repaired/restored by 24 h. However, the most severe degree of mtDNA damage, elicited by the highest concentration of GOx used (0.009 U/ml) induced bioenergetic alterations that were no longer reversible at 24 h. There were marked increases in the expression of a multiple pro-inflammatory mediators IL-1α, IL-1β, IL-6, IL-8, TNFα, and VGFA and reduced expression of MCP1 in response to mtDNA-specific damage at 24 h post GOx-treatment (Fig. 2G). The amount of inflammatory mediator production was proportional with the degree of the oxidative stress applied. In a control experiment, heat inactivated GOx (15 min at 65 °C) failed to induce cytokine production (Fig. 2H).

To further confirm the link between mtDNA-specific damage and inflammation, we used lung adenocarcinoma cells (A549) and their mtDNA-depleted variant (A549 rho0) (Fig. S1J). When challenged with GOx, A549 cells responded with enhanced expression of IL-6, while in A549 rho0 no oxidative-stress-induced increase in inflammatory mediator production was detected, confirming that the presence of the mtDNA is necessary to induce inflammation (Fig. S1J). Taken together, these data indicate that prolonged, low-level oxidative stress, in the absence of any detectable loss of cell viability (neither necrosis nor apoptosis), induces mtDNA-specific damage, which is associated with a transient remodeling of cellular bioenergetics and morphology, and with the production of multiple pro-inflammatory mediators.

### Damaged mtDNA binds to Z-DNA binding protein 1 (ZBP1) and induces inflammation

**Figure 3:**
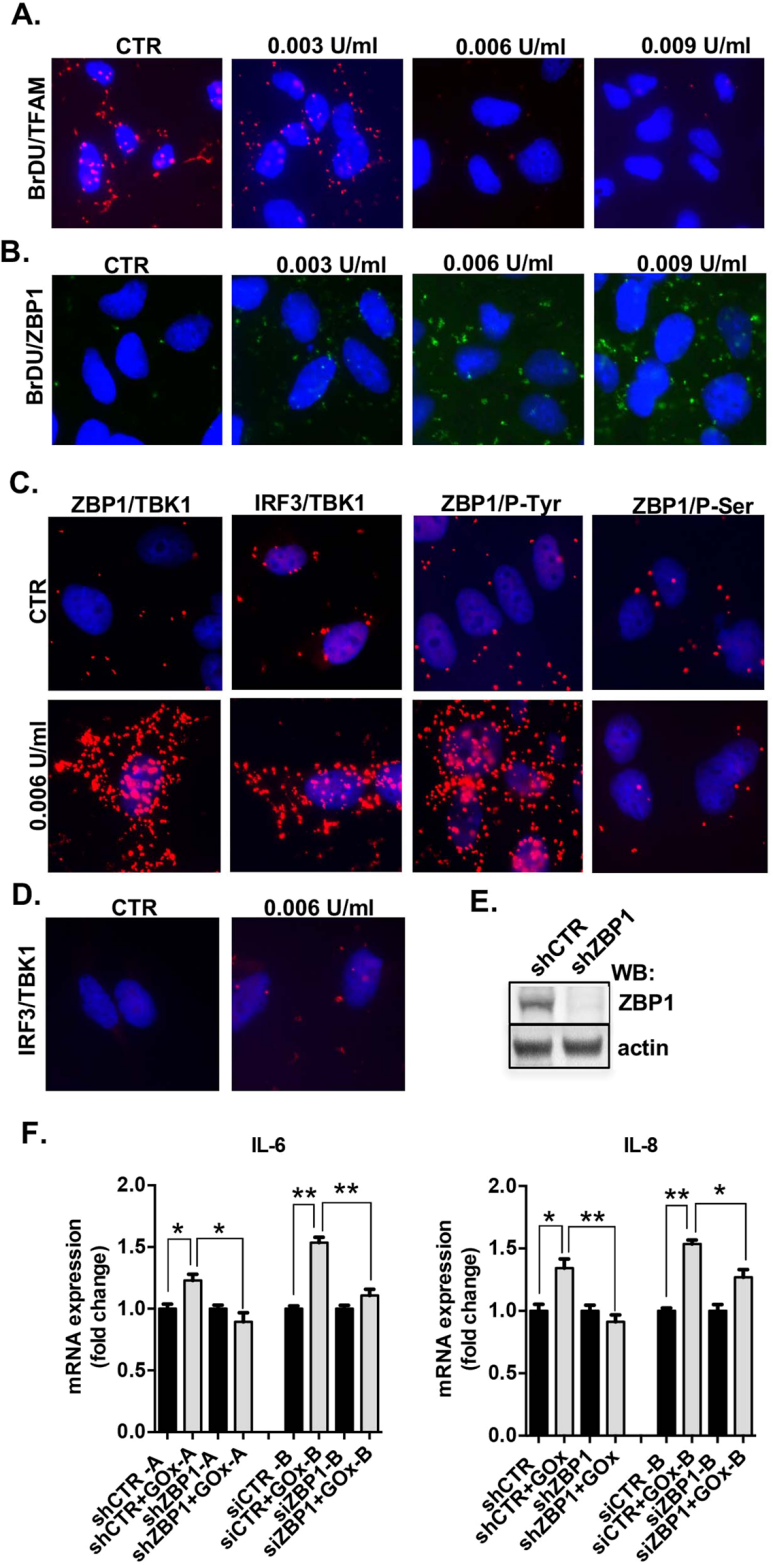
Damaged mitochondrial DNA activates ZBP1/TBK1/IRF3 signaling pathway. Interaction between BrDU-labelled mtDNA and TFAM (**A**) and BrDU-labelled mtDNA and ZBP1 (**B**) in control and GOx-treated cells at 1 h. (**C**) Interaction between ZBP1/TBK1, IRF3/TBK1, ZBP1/P-Tyr and ZBP1/P-Ser in control and GOx-treated cells at 1 h. (**D**) Interaction between IRF3/TBK1 in pre-treated with 3 μM of CsA in GOx-treated BEAS 2B cells. (**E**) Level of ZBP1 depletion, shown by Western blotting. (**F**) Expression of IL-6 and IL-8 in unstressed and GOx-treated (0.006 U/ml for 1 h) in control and ZBP1-depleted BEAS2B cells. Representative images of n = 3 independent experiments are shown. Data represent average ± SEM of n = 5 biological replicates. *p < 0.05 *vs*. control.

Next, we investigated the signaling pathway(s) linking mtDNA damage to the expression of pro-inflammatory mediators. Mammalian cells have many extra- and intracellular DNA and RNA sensing receptors^29,30^. Since replication of mtDNA is cell cycle independent and contact-inhibited cells do not replicate their nuclear DNA, we specifically labeled mtDNA with BrDU in confluent BEAS 2B cells. Using a proximity ligation assay (PLA), we investigated *in cellulo* binary interaction between BrDU and various putative DNA-binding targets. The assay was validated with mitochondrial transcription factor A (TFAM), a protein that stably interacts with mtDNA in physiological condition^31^. Interaction between BrDU-labelled mtDNA and TFAM was observed only in control cells (Fig. 3A). Next, we surveyed the known DNA-specific receptors for recognition of damaged mtDNA and detected an interaction between the cytosolic DNA sensor Z-DNA binding protein 1 (ZBP1) and BrDU-labeled damaged mtDNA in response to GOx-treatment (Fig. 3B). No interaction was not found with any of the other tested DNA-sensors: TLR9, AIM2, NLRP3 and cGAS (Fig. S3A).

Activation of ZBP1 is known to induce recruitment of TANK-binding kinase 1 (TBK1) and interferon regulatory factor 3 (IRF3), with a subsequent phosphorylation of ZBP1^32,33^. In response to GOx-treatment, we detected a significant enhancement of the interaction between ZBP1/TBK1, IRF3/TBK1 and phosphorylation of ZBP1 on Tyr, but not on Ser residues (Fig. 3C). This finding confirms the activation of ZBP1/TBK1/IRF3 signaling pathway in response to mtDNA-specific damage. The interaction between TBK1 and IRF3 was markedly reduced in GOx-treated cells pre-treated with cyclosporine A (CsA), an inhibitor of mitochondrial permeability transition pore, suggesting that the release of the mtDNA from mitochondria is involved in the process of TBK1/IRF3 activation (Fig. 3D). Next, ZBP1 was stably (shRNA-A) and transiently (siRNA-B) silenced with specific RNAi and GOx-induced pro-inflammatory cytokine production was measured (Figs 3E and S3B). As expected, in control (scrambled RNA) treated cells, GOx induced inflammatory cytokine mRNA production (IL-6, IL-8), while in ZBP1-silenced cells, this response was absent (Fig. 3F). Taken together, these data show that damaged mtDNA induces inflammation *via* activation of the ZBP1/TBKI/IRF3 pathway.

### Damaged mtDNA is released from the cells *via* exosomes

**Figure 4:**
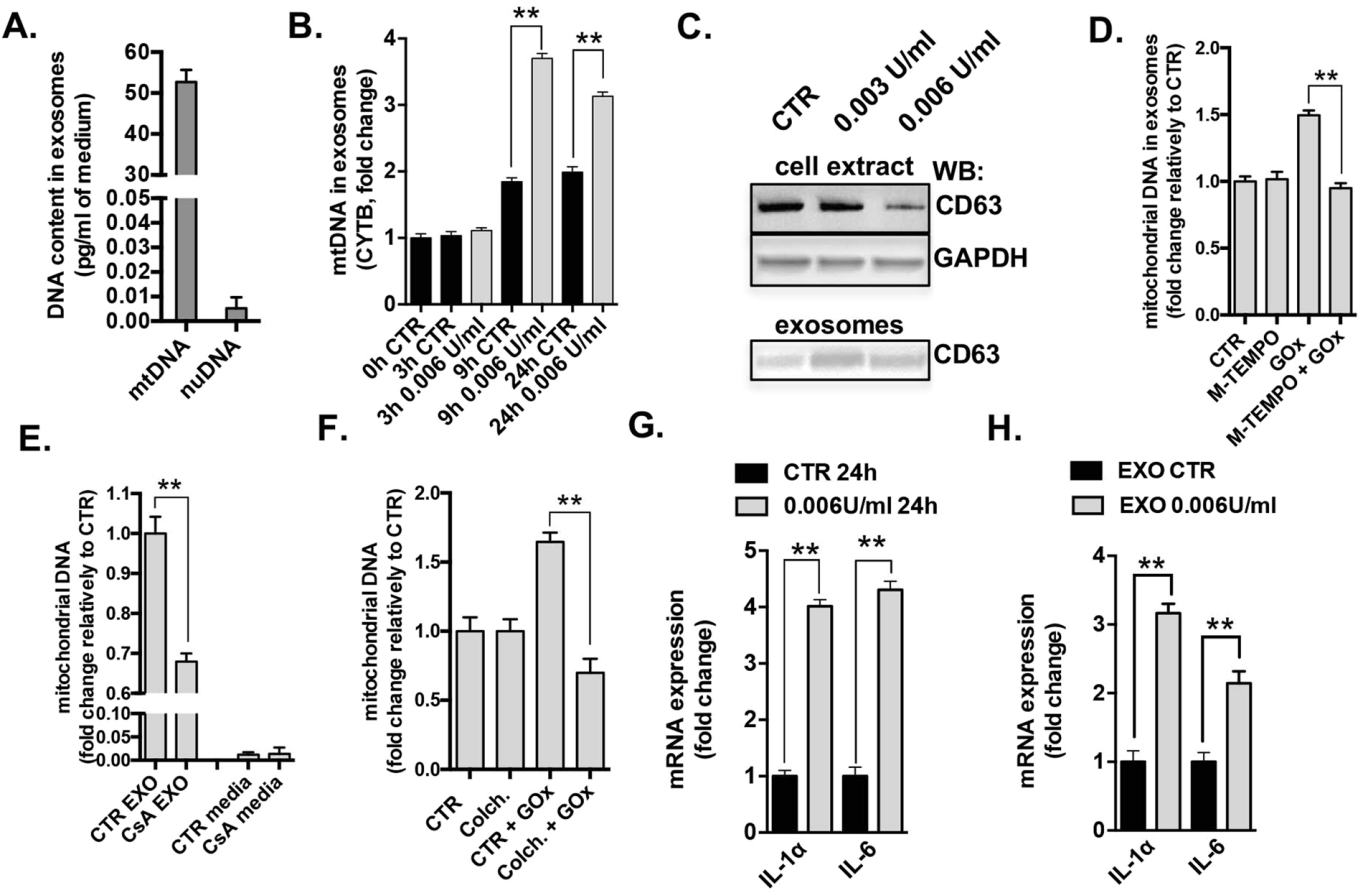
Oxidatively stressed cells induce release of damaged mtDNA *via* exosomes. (**A**) Comparison of mitochondrial and nuclear DNA in exosomal fraction isolated at 24 h from BEAS 2B cells treated with 0.006 U/ml GOx. (**B**) Time-dependent release of the mtDNA from control and BEAS 2B cells treated with 0.006 U/ml of GOx. (**C**) Protein level of CD63, marker of exosomes, in BEAS 2B cell extract and in exosomal fraction. MtDNA release from cells pretreated for 1 h with (**D**) 300 nM of Mito-TEMPO; (**E**) 3 μM cyclosporine-A or (**F**) 3 μM colchicine in control and 0.006 U/ml of GOx-treated BEAS 2B cells. Data represent average ± SEM of n = 5 biological replicates. Representative images of n = 3 independent experiments are shown. **p < 0.01 *vs*. control.

Because oxidative stress resulted in the cellular depletion of mtDNA at 24 h (Fig. 2B), next we investigated whether mtDNA is released from GOx-stressed cells. Circulating microvesicles are emerging as important players in cell-to-cell communication^34^. The analysis of nanoparticles in cultured medium at 24 h post-treatment did not show any significant changes in amount and sizes between control and GOx-treated cells, but the abundance of nanoparticles of 100–150 nm indicated the presence of exosomes (Fig. S4). Analyzing the isolated exosomal fraction, we detected significant amounts of mitochondrial (but not nuclear) DNA in the exosomes of cells subjected to GOx treatment (Fig. 4A). The presence of mtDNA in the exosomal fraction was further confirmed by analysis of three different regions of the mtDNA encoding NAD, CYTB, COXIII subunits (Fig. S5A). The amount of mtDNA in the exosomal fraction correlated with the degree of the mtDNA damage (Fig. S5A). The culture medium itself, after exosomal isolation, did not contain any significant amount of mtDNA (Fig. S5A), suggesting a specific, active mechanism of extracellular extrusion of the damaged mtDNA. Interestingly, we also noted a low level, time-dependent accumulation of mtDNA in exosomes in unstressed cells (Fig. 4B), possibly as a consequence of intrinsic oxidative stress or perhaps physiological horizontal transfer of mtDNA. The release of exosomes from oxidatively stressed cells was further confirmed by demonstration of cellular depletion of the exosomal marker CD63^35^ and its increase in the exosomal fraction (Fig. 4C). Treatment of BEAS 2B cells with Mito-TEMPO, a mitochondrial specific antioxidant, abolished the release of mtDNA to the exosomes (Fig. 4D), suggesting that mitochondrial oxidative stress, on its own, is a sufficient stimulus to initiate mtDNA release. CsA or colchicine, an inhibitor of microfilament organization (a pharmacological tool to interrupt intracellular microvesicle transportation); both reduced the release of mtDNA in the GOx-treated cells (Fig. 4E,F). Analysis of exosomal content indicated relatively large (<3,000 bp) fragments of mtDNA in exosomes isolated from GOx-treated BEAS 2B cells (Fig. S5B).

Finally, we investigated whether the exosomal fraction which contains the damaged mtDNA is capable of inducing an inflammatory response in unstressed (naïve) cells. Exosomes were isolated from the culture medium of control and GOx treated cells (Fig. 4G) and were subsequently placed onto unstressed cells. There was an enhanced expression of IL-6 and IL-8 in the cells treated with exosomal fractions containing oxidatively damaged mtDNA (Fig. 4H). The oxidative stress induced mtDNA depletion (Fig. S6A) as well as the release of mitochondrial, but not nuclear, DNA via exosomes (Fig. S6B,C) were confirmed in a second cell line (A549 lung adenocarcinoma cells), suggesting that oxidative stress-induced release of mtDNA is a generalized mechanism in pulmonary epithelial cells.

### Damaged mtDNA induces inflammation in mouse models

**Figure 5:**
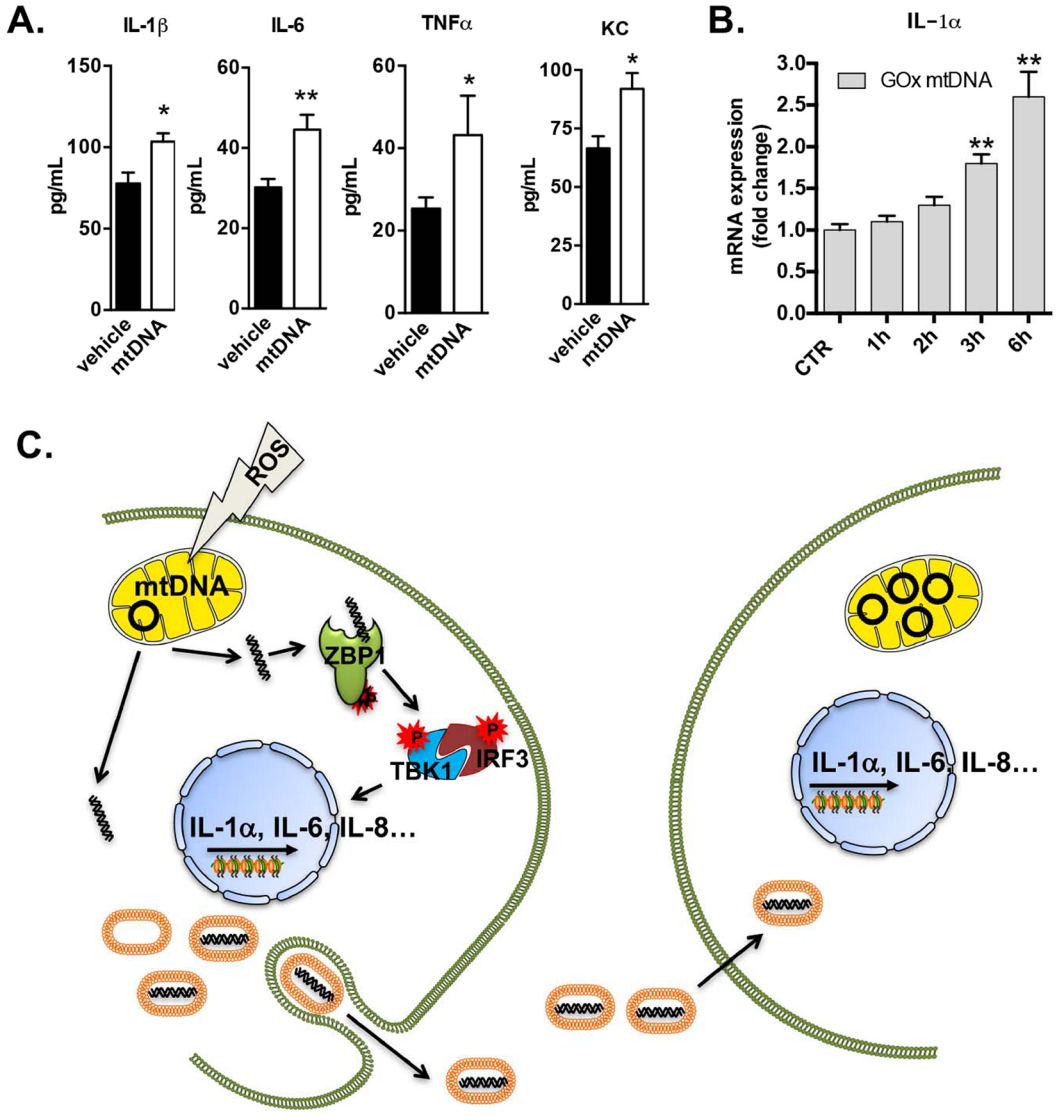
Inflammation respond to mitochondrial DNA *in vivo*. (**A**) Inflammatory response triggered in BALF of naïve mice at 24 h post intratracheal administration of 1 μg of isolated mtDNA. (**B**) Time-dependent expression of IL-1α in BEAS 2B cells incubated 100 ng of isolated mtDNA. (**C**) Model of inflammation induced by mtDNA/ZBP1 pathway. Data represent average ± SEM using n = 6–8 animals or n = 3 for biological replicates per experimental end-point. *p < 0.05, **p < 0.01 *vs*. control.

Since isolation of exosomal fractions containing mtDNA from mouse tissue is not feasible, we examined whether intra-tracheal administration of isolated mtDNA can induce a pulmonary inflammatory response in naïve mice. MtDNA of various sizes was isolated from mouse liver (Fig. S7A). At 24 h post-administration of large mtDNA fragments, significant increases in IL-1β, IL-6, TNFα, KC and VEGF were detected in the BALF of treated mice (Fig. 5A). No increases were noted in levels of IL-2, IL-4, IL-5 and IP-10 (Fig. S7B), and injection of mtDNA did not affect gross pathology of the lungs (Fig. S7C,D). Moreover, injection of large and small mtDNA fragments (Fig. S7A) resulted in comparable inflammatory response. The profile of induced pro-inflammatory mediators was similar to that detected in GOx-treated epithelial cells (Fig. 2G). Finally, in order to confirm the induction of inflammatory response induced by isolated mtDNA fragments (without exosomal “packaging”), we measured a time-dependent enhanced expression of the IL-1α in cultured BEAS 2B cells (Fig. 5B). Taken together, our *in vivo* studies mirror our *in vitro* observations and indicate that mtDNA release occurs in response to oxidative stress and it is capable of inducing an inflammatory response - all without any detectable evidence of cell death or histopathological abnormalities.

## Discussion

This study identifies a role for active and selective cellular extrusion of damaged mitochondrial (but not nuclear) DNA in response to prolonged, low-level oxidative stress in pulmonary epithelial cells *in vitro*. Damaged mtDNA first enters the cytoplasm where it interacts with ZBP1 to trigger inflammatory signaling via activation of the TBK1/IRF3 signaling pathway. Damaged mtDNA is also extruded from cells via exosomes, and triggers inflammation in unstressed, naive cells. These processes occur in fully viable cells that are subjected to survivable levels of mtDNA-specific damage. Evidence for extrusion/inflammation and concomitant damage/depletion of mtDNA in lung tissue was also provided in a mouse model *in vivo*. The extrusion of damaged mtDNA and the induced downstream signaling both represent active, regulated processes in cells exposed to extended, low-level oxidative stress. These processes may induce and amplify the inflammatory response in autocrine as well as paracrine manner (Fig. 5C). We propose that the release of damaged mtDNA acts as a functional link between low-level oxidative stress and chronic inflammation in parenchymal cells and we hypothesize that this process may contribute to the development of various pulmonary inflammatory diseases.

There are three major findings of our report: 1) demonstration of the active and selective release of mtDNA in response to low-level oxidative stress by viable (non-necrotic/non-apoptotic) epithelial cells; 2) identification of ZBP1 as an intracellular receptor recognizing damaged mtDNA; and 3) demonstration that low-level oxidative stress, and consequent mtDNA-specific damage can induce inflammation in epithelial cells. Recent studies showed that endogenous molecules (including DNA) are released during tissue injury or cell damage and can trigger inflammatory response by binding to receptors that typically recognize DNA from viral or bacterial pathogens. This mechanism is designated as “sterile inflammation” and it is induced by damage-associated molecular patterns (DAMPs). In prior studies, these processes were characterized in the context as consequences of severe cell injury (cell necrosis) and involved the release of mixed cellular content (nuclear DNA and nuclear proteins; mtDNA and mitochondrial proteins, etc.)^36,37^. The first suggestion that mtDNA may induce an inflammatory response comes from studies by Collins, who reported that ectopic administration of isolated mtDNA can induce arthritis^38^. Hauser’s group subsequently showed that mitochondrial DAMPs, including formyl peptides and mtDNA, activate human neutrophils via the formyl peptide receptor and TLR9, respectively^11^. Oxidized mtDNA was found to bind to the NLRP3 inflammasome in apoptotic macrophages, inducing IL-1β production^15,16^. Release of mtDNA from mitochondria during intrinsic cell death induces IFNγ expression via the cGAS/STING pathway^12–14^. Several groups have previously investigated the presence of mtDNA in body fluids in various conditions, including in synovial fluids of rheumatoid arthritis patients^16^ and in the plasma of mice subjected to hemorrhagic shock^39^ or burn injury^40^, among others^10^. These prior processes are in stark contrast to the mechanisms outlined in this report, where selective mtDNA damage occurs, followed by its regulated release in response to low-level oxidative challenge, from which the cells can recover. In contrast to the previously studied pathways, the current scenario identifies ZBP1 as a cytoplasmic DNA-sensor recognizing mtDNA. ZBP1 was initially implicated in the binding of the double stranded B-DNA form, with downstream signaling through NF-κB, TBK1 and IRF3^32,33^. All prior reports implicated ZBP1 as a sensor to foreign DNA; however, our data indicate endogenous oxidatively damaged mtDNA can also bind to ZBP1 to promote inflammation. The results of lung injury induced in the cigarette smoke model suggest that early release of mtDNA also occurs in response to oxidative stress *in vivo*.

Based on results shown in Fig. 2, cells with low mtDNA content can be fully functional, as judged by their oxidative phosphorylation parameters. This functionality holds true as long as the small amount of remaining mtDNA is intact. At later time points, it is likely that mtDNA will replicate itself and its content will recover. The pro-inflammatory response signaling, which accompanies mtDNA release, may either be an unintended “byproduct” of the extrusion process or perhaps may serve as a damage signal to prepare the cell for noxious environmental conditions. We hypothesize that pharmacological inhibition of ZBP1 may be a potential future approach to reduce inflammation in cells exposed to chronic low-level oxidative stress.

The exact biochemical nature of the extruded damaged mtDNA remains to be characterized. Oxidative stress induces multiple types of DNA lesions, including strand breaks and base modifications^41^, thus remains unknown whether ZBP1 recognize particular DNA lesion or fragments of linear mtDNA. Also, the exact mechanism of how damaged mtDNA is released to the cytoplasm requires additional analysis. Since mtDNA encodes key elements of the mitochondrial electron transport chain, we speculate that the physiological reason for extrusion of damaged mtDNA may be to prevent transcription and translation of dysfunctional mitochondrial proteins. Taken together, based on the findings of the current report, we suggest that mtDNA-specific damage and ZBP1 activation link oxidative stress to inflammation, and these mechanisms may contribute to the pathogenesis of various inflammatory lung diseases.

## Methods

### Cell culture and treatments

BEAS2B cells (ATCC CRL-9609) were maintained in RPMI 1640 medium supplemented with 10% FBS at 37 °C in 5% CO_2_. To induce oxidative stress, cells were treated with fresh medium containing GOx (Sigma-Aldrich G2133) for 1 h, followed by two washes with medium to remove GOx. Cells were either analyzed immediately at 1 h or were maintained in RPMI 1640 medium for additional 24 h. We have shown before that e.g. 0.003 U/ml of GOx generates within 1 h similar amount of hydrogen peroxide as 30 μM H_2_O_2_^42^.

### Animal models

Studies in C57BL/6 J mice (eight to ten weeks old) were conducted in compliance with the Principles of Laboratory Animal Care formulated by the National Society for Medical Research. To induce cigarette smoke injury, mice were exposed to dilute mainstream cigarette smoke using an automated smoking machine as previously described^27^. Mice were exposed to ten cigarettes (3R4F University of Kentucky, KY); control group was exposed to air. Animals were sacrificed with desflurane. The trachea was cannulated and lungs lavaged with 3 × 0.5 ml saline. BALF was centrifuged in 1000 × g for five min at 4 °C. Supernatant was collected and snap-frozen in liquid nitrogen. Lung tissue was analyzed for DNA integrity and content.

For intratracheal injection of vehicle (50 μl TE) or mtDNA (1 μg in 50 μl TE), mice were treated with a s.c. injection of buprenorphene and anesthetized with 1–3% isoflurane gas. At 24 h post injection BALF was collected. MtDNA was isolated from mouse liver by using Mitochondrial DNA Isolation Kit (abcam, ab65321). Prior injection, mtDNA was analyzed on 1% agaroze gel. Sonication was used to generate mtDNA fragments. Lungs were analyzed for histology and for MPO activity as before^40^. This animal studies were approved by the Institutional Animal Care and Use Committee (IACUC) of the University of Texas Medical Branch and were carried out in accordance to the approved guidelines.

### DNA damage

Measurement of mitochondrial and nuclear DNA integrity was conducted using gene-specific semi-quantitative PCRs as described^28^. Briefly, BEAS 2B cells were seeded in 12 well plates (100,000 cell/well). DNA was isolated immediately after GOx treatment and 24 h later using DNasey Blood and Tissue Kit (QIAGEN) according to manufactures recommendations. Integrity of the mtDNA was assessed using two set pairs of primers with amplicon of 211 bp and 8.9 kb (short amplicon was used as a normalization factor of mtDNA copies)^28^. Integrity of the nuclear DNA was assessed using 10 kb amplicon. *Taq* DNA Polymerase and LongAmp *Taq* DNA polymerase (both from NewEngland BioLabs) were used for amplification of short and long DNA targets, respectively. DNA integrity in untreated (control) cells was set as 100%.

### Apoptotic and necrotic cell death

Briefly, BEAS 2B cells were seeded in 6 well plate (10^6^ cells/well). The amount of apoptotic and necrotic cell death was assessed at 1 h and 24 h post GOx-treatment using the PE Annexin V Apoptosis Detection Kit (BD Biosciences) according to the manufacturer’s instructions. 10^4^ events were sorted and quantified using the EasyCyte™ Plus Flow Cytometer (Guava). Early apoptotic (AnxV+/7-AAD−), late apoptotic (AnxV+/7-AAD+), and necrotic (AnxV−/7-AAD+) populations. Camptothecin (10 µM) was used as a positive control.

### Cellular bioenergetics

Analysis of mitochondrial respiration and glycolytic function was assessed using the XF24 Extracellular Flux Analyzer (Agilent). Briefly, BEAS 2B cells were seeded on cell culture microplates (60,000/well) 24 h prior treatment/analysis. For analysis of mitochondrial respiration, cells were washed twice with low glucose DMEM medium pH 7.4 supplemented with L-Glutamine (2 mM, GIBCO) and sodium pyruvate (0.33 mM, SIGMA). After 1 h incubation at 37 °C in CO_2_ free incubator, the oxygen consumption rate (OCR) after oligomycin (1.5 µg/mL) was used to assess ATP production rate and the OCR after carbonyl cyanide-4-trifluoromethoxy phenylhydrazone (FCCP, 0.5 µM) to assess maximal mitochondrial respiratory capacity. Antimycin A (2 µg/mL) and rotenone (2 µM) were used to inhibit the flux of electrons through complex III and I, to detect residual non-mitochondrial OCR, which is considered to be due to cytosolic oxidase enzymes. For analysis of glycolytic parameters, cells were washed twice with DMEM medium without glucose. After 1 h incubation at 37 °C in CO_2_ free incubator, the extracellular acidification rate (ECAR) after addition of glucose (10 mM) was used to asses glycolysis and the ECAR after addition of oligomycin (1.5 µg/mL) was used to determine glycolytic capacity. 2-deoxyglucose (100 mM) was used to inhibit glycolysis and asses non-glycolytic acidification.

### Fluorescent microscopy

Cells were fixed and permeabilized with 4% paraformaldehyde, 0.2% Tween-20 in PBS from 15 min. After three washes with PBS, cells were permeabilized with PBS containing 0.25% Triton X100 for 15 min. Samples were incubated in 5% BSA blocking solution for 1 h and stained with mouse anti-ATPB (Abcam [3D5] ab14730, 2 µg/ml) and rabbit anti-β-tubulin (Cell Signaling #2146, 1/50) overnight at 4 °C. After 3 PBS washes, cells were probed with mouse and rabbit-specific fluorescent-labeled secondary antibodies (1:200, Alexa Fluor 488 and 546, Life Technologies) for 2 h. Slides were washed with PBS, air-dried and mounted with DAPI containing solution. Fluorescent signal was visualized using the confocal inverted microscope Nikon Eclipse T*i* customized by Praire Technologies (Bruker Corp., Billerica, MA). Analyses were performed using multi-channels configuration with a 100 X objective and images captured with Camera Quantem 512*SC* (Photometrics, Tucson, AZ). IMARIS 8 Software was used for 3D-reconstruction of the mitochondrial network.

### Quantification of cytokine/chemokine levels

BEAS 2B cells were plated in 12 well plates (200,000/well) followed by incubation at 37 °C, 5% CO_2_ humidified incubator overnight. Amount of cytokines/chemokines was analyzed in supernatant of cells or in mice BALF was analyzed with Human/Mouse Cytokine/Chemokine Magnetic Bead Panel (EMD Millipore) according to manufacture’s recommendations using the Luminex system.

### Quantitative real-time PCR

BEAS 2B cells were seeded in 6 well plates (10^6^ cells/well). Next day, cells were treated with GOx and at 1 h and 24 h post-treatment the DNA from the exosomal fraction of the supernatant (1 ml) or cell extract was isolated with DNasey Blood and Tissue Kit (QIAGEN). To quantify DNA in exosomes, we used the CFX96 TouchTM Real-Time PCR Detection System (Bio-Rad) and Maxima SYBR Green/ROX qPCR Master Mix (Thermo Scientific) with the following primers: *CYTB*: FW 5′-ATGACCCCAATACGCAAAAT-3′, RV 5′-CGAAGTTTCATCATGCGGAG-3′; *NAD1*: FW 5′-ATACCCATGGCCAACCTCCT-3′, RV 5′-GGGCCTTTGCGTAGTTGTAT-3′; *COXIII*: FW 5′-TGACCCACCAATCACATGC-3′, RV 5′-ATCACATGGCTAGGCCGGAG-3′; *SIRT1*: FW 5′-CCCGCAGCCGAGCCGCGGGG-3, RV 5-TCTTCCAACTGCCTCTCTGGCCCTCCG-3′.We used the following thermal cycle: 95 °C for 10 min, 40 cycles at 95 °C for 15 s, and 60 °C for 1 min. Expression of mtDNA-specific genes (*CYTB*, *NAD1*, *and COXIII*) was compared with the expression of the nuclear gene (*SIRT1)*.

### RNA extraction, qRT-PCR

Total RNA was isolated from BEAS2B cells using the RNeasy Mini Kit (Qiagen) and reverse transcribed with the High-Capacity cDNA Reverse Transcription Kit (Applied Biosystems). The cDNA was used for qPCR analysis using the Maxima SYBR Green/ROX qPCR Master Mix (Thermo Scientific) and CFX96 TouchTM Real-Time PCR Detection System (Bio-Rad) with the following primers: *IL-1A*: FW 5′-AGTAGCAACCAACGGGAAGG-3′, RV 5′-TTCCTCTGAGTCATTGGCGA-3′; *IL-6*: FW 5′-AGTGAGGAACAAGCCAGAGC-3′, RV 5′-ATTTGTGGTTGGGTCAGGGG-3′; *IL-4*: FW 5′-CAACTGCTCCCCCTCTGTT-3′, RV 5-TCTGCTCTGTGAGGCTGTTC-3′; *GADH*: FW 5′-GCACCACCAACTGCTTAGC-3′, RV 5′-GGCATGGACTGTGGTCATGAG-3′; using the following cycle: 95 °C for 10 min, 40 cycles at 95 °C for 15 s, and 60 °C for 1 min. The expression of the interleukins was normalized to *GAPDH*.

### Isolation of exosomes

BEAS 2B cells were seeded in 6 well plates (10^6^ cells/well) and next day treated with GOx. At 1 h and 24 h post-treatment exosomal fraction was isolated from the culture medium (1 ml) using the Total Exosome Isolation kit (Invitrogen, 4478359). Exosomal pellet was lysed with SDS-sample buffer for Western analysis or in PBS for total DNA isolation (DNeasy Blood and Tissue Kit, QIAGEN), followed by determination of mitochondrial and nuclear DNA by qPCR. In these experiments, we used exosome-free FBS.

### ZBP1 depletion

ZBP1 was stably and transiently depleted from BEAS 2B cells. For stable ZBP1 depletion we used shRNA specific for human ZBP1 (Origene, TL300389V). Briefly, 10^5^ cells/well were seeded in 6 well plates. Next day, (70–80% confluence) based on number of cells we calculated the amount of viral particles to use in order to achieve Multiplicity Of Infection (MOI) of 4. We used Lipofectamine 2000 (Thermo Fisher Scientific) as transfection reagent and the polybrene (8 μg/ml) to increase the efficiency of the transfection. After 5 days post infection, we used 0.5 μg/ml of puromicyn (Thermo Fisher Scientific) for cell selection. The level of depletion was verified by Western analysis once the selection was completed (about 3–4 days) and checked later for each experiment. For transient ZBP1 depletion we used siRNA construct specific for human ZBP1 (Origene, SR312989B). Briefly, 3 × 10^5^ cells/well were seeded in 6 well plates and the next day they were transfected with 30 nM siRNA following the protocol of Lipofectamine RNAiMAX (Thermo Fisher Scientific). At 36 hours post transfection BEAS 2B cells were treated with GOx (0.006U/ml) and expression of inflammatory markers were checked at 24 h.

### Proximity Ligation Assay (PLA)

20,000 of BEAS 2B cells were seeded in Lab-Tek Chamber Slide w/Cover (Thermo Scientific) followed by incubation at 37 °C, 5% CO_2_ humidified incubator overnight. Cells were fixed and permeabilized with 4% paraformaldehyde, 0.2% Tween-20 in PBS from 20 min. After three washes with PBS, PLA was performed according to manufacturer’s instructions using Duolink *In Situ* Kit (SIGMA) with following a pair of primary antibodies raised in two different species: BrDU (mouse; SigmaAldrich), TFAM (rabbit; GeneTex), ZBP1 (rabbit; Protein Tech), TLR9, AIM2, NLRP3 and cGAS (all rabbit; Cell Signaling). Images were captured using Nikon eclipse 80i inverted fluorescent microscope with Photometric CoolSNAP HQ2 camera and NIS-Elements BR 3.10 software. The interaction between proteins (or proteins phosphorylation) was visualized using green or red fluorescent signal.

### Statistical analysis

All *in vitro* experiments were performed in at least three biological replicates. Six to eight animals/group were used for *in vivo* studies. All data are presented as means ± SEM and were analyzed using GraphPad Prism software. Statistical analyses included Student-t test or one-way ANOVA followed by Bonferroni’s multiple comparisons.

## Electronic supplementary material

Supplementary information
