# Supplementary material for: Mitochondrial DNA damage and subsequent activation of Z-DNA binding protein 1 links oxidative stress to inflammation in epithelial cells
Source: Sci Rep. 2018 Jan 17;8:914. doi: 10.1038/s41598-018-19216-1 (PMC5772643; doi:10.1038/s41598-018-19216-1)
Supplement: Supplementary file 1 — Supplementary information [file 41598_2018_19216_MOESM1_ESM.pdf]

# Mitochondrial DNA damage and subsequent activation of Z-DNA binding protein 1 links oxidative stress to inflammation in epithelial cells

Bartosz Szczesny[1*], Michela Marcatti[1], Akbar Ahmad[1], Mauro Montalbano[2], Attila Brunyánszki[1], Sofia-Iris Bibli[3], Andreas Papapetropoulos[3,4], and Csaba Szabo[1*]

[1]Department of Anesthesiology, University of Texas Medical Branch, Galveston, TX, USA
[2]Center for Biomedical Engineering, University of Texas Medical Branch, Galveston, TX, USA
[3]Faculty of Pharmacy, University of Athens, Athens, Greece
[4]Center of Clinical, Experimental Surgery & Translational Research, Biomedical Research Foundation of the Academy of Athens, Greece

[*]Corresponding authors: baszczes@utmb.edu (B.S) or szabocsaba@aol.com (C.S).

Running title: Inflammation mediated by ZBP1/mtDNA axis

**Supplementary information**

**Supplementary Methods**

**Intracellular oxidative stress** induced by GOx-treatment was assayed with CM-$H_2$DCFDA (Molecular Probes, Invitrogen). Briefly, 30,000 BEAS 2B cells per well were seeded in 96 well optical black cell culture plates. After overnight incubation at 37$^o$C, 5% $CO_2$ in humidified incubator, the cells were loaded with 10 μM of CM-H2DCFDA dye for 30 min. At various time points, fluorescence was measured with SpectraMax M2 microplate reader (Molecular Devices).

**Lactate dehydrogenase release** was used as a cytotoxicity assay to measure permeability of the plasma membrane. Briefly, 30 µl of supernatant was saved from BEAS 2B cells treated with GOx and mixed with 100 µl of freshly prepared LDH assay reagent to reach final concentrations of 85 mM lactic acid, 1040 mM nicotinamide adenine dinucleotide ($NAD^+$), 224 mM *N*-methylphenazonium methyl sulfate (PMS), 528 mM 2-(4-Iodophenyl)-3-(4-nitrophenyl)-5-phenyl-2H-tetrazolium chloride (INT) and 200 mM Tris (pH 8.2). The changes in absorbance were read kinetically at 492 nm for 15 min (kinetic LDH assay) on a monochromator-based reader (Powerwave HT, Biotek) at 37 ºC. LDH activity values are shown as $V_{max}$ for the kinetic assays in mOD/min.

**Citrate synthase activity,** an indicator of the mitochondrial volume, was measured with a Citrate Synthase Assay Kit (Sigma-Aldrich, CS0720). Briefly, total cell extract was prepared using NP-40 lysis buffer (100 mM NaCl, 50 mM Tris-Cl pH 8.0, 1 % NP-40). 10 µg of cell extract was used for determination of citrate synthase specific activity by measuring amount of 5-thio-2-nitrobenoic acid (TNB, yellow product) from reaction of DTNB with thiol-containing acetyl coenzyme A produced from acetyl coenzyme A and oxaloacetate by citrate synthase.

**Mitochondrial membrane potential** was measured using the TMRE-Mitochondrial Membrane Potential Assay kit (Abcam, ab113852).

**MitoSOX Red staining**, a mitochondrial superoxide indicator (Invitrogen M36008) was used to detect mitochondrial ROS production. Briefly, 40,000/well of BEAS 2B cells were seeded in Lab-Tek II chamber slides (Nalgen Nunc International) and incubated at 37$^o$C, 5% $CO_2$

humidified incubator overnight. The cells were loaded with 5 μM of MitoSOX Red dye (Invitrogen M36008) for 10 min. For analysis, cells were washed three times with PBS and dye's specific fluorescence was visualized using Nikon eclipse 80i inverted microscope with Photometric CoolSNAP HQ2 camera and NIS-Elements BR 3.10 software.

**Nanoparticle tracking analysis (NTA).** NTA measurements were performed for size and concentration of nanoparticles using a NanoSight NS300 instrument (NanoSight, Malvern Instruments, USA), following the manufacturer's instructions. Medium of BEAS 2B cells were diluted to reach a particle concentration suitable for analysis with NTA ($10^6$ to $10^9$ particles/ml). Two different dilutions for each sample were prepared and analyzed each one three times. The samples were injected into the NS300 unit (approximately 300 μl) with a 1 ml sterile syringe. The capturing settings (shutter and gain) and analyzing settings were manually set according to the protocol suggested in the technical note and then optimized for specific nanoparticles. The NanoSight NS300 recorded 60 second sample videos which were than analyzed with the Nanoparticle Tracking Analysis (NTA) 2.0 Analytical software. Size/concentration PDF reports and videos were obtained for each set of data (5 fields recorded for each sample). The average of all 6 reads per sample are shown.

**Mitochondrial DNA size analysis** in exosomal fraction was conducted with the 2100 expert Bioanalyzer (Agilent) using the Agilent 1200 kit. Briefly, $10^6$ cells/well were seeded in 6 well plates. Next day cells were treated with GOx and at 24 h post-treatment total DNA from the exosomal fraction of the supernatant (1 ml) was extracted using DNasey Blood and Tissue Kit (QIAGEN) and analyzed according to manufacturer's recommendations.

# Supplementary Figure Legends

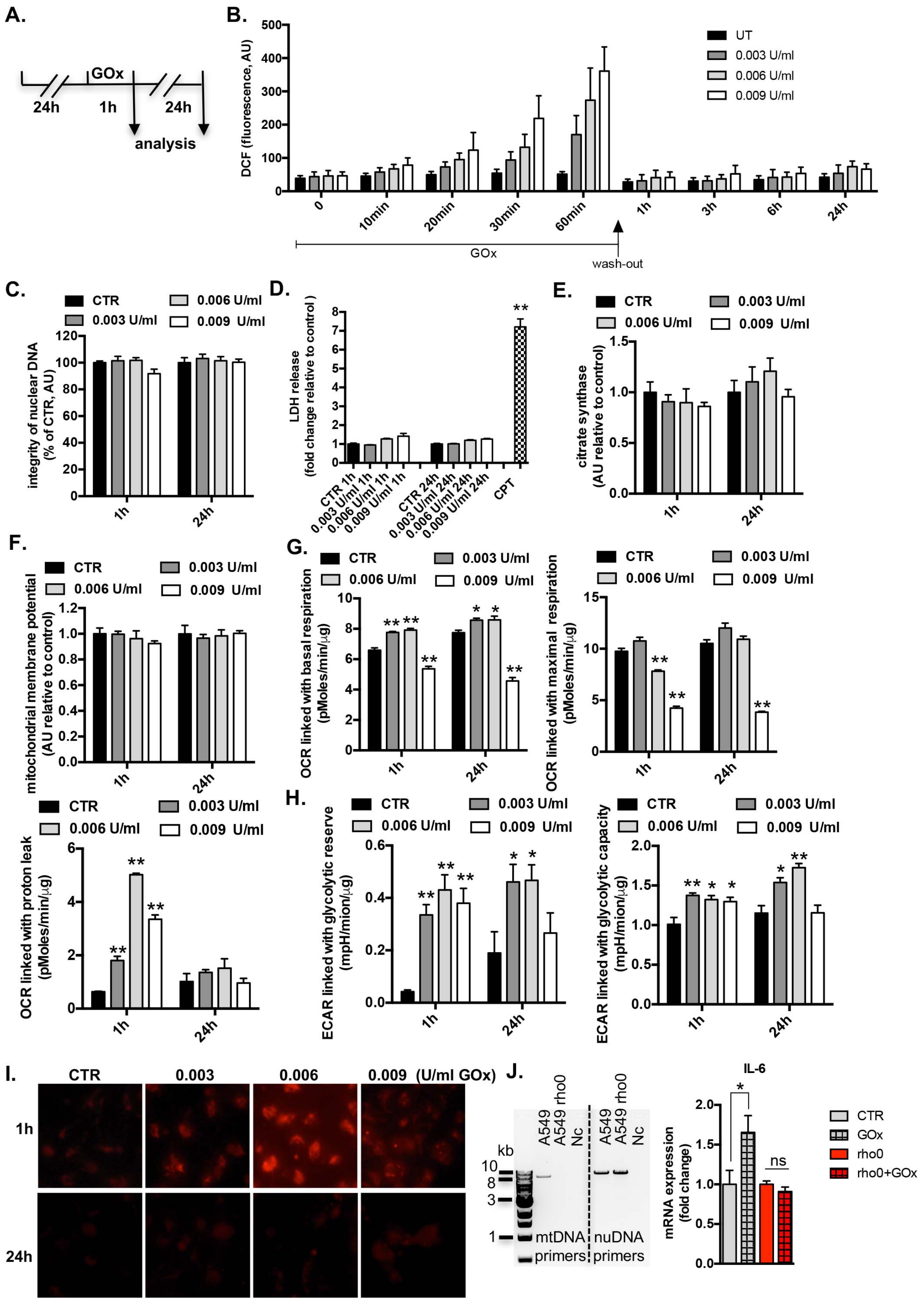

**Fig. S1**. **Non-cytotoxic level of oxidative stress does not induce nuclear DNA damage, impairment of plasma membrane integrity, changes in mitochondrial volume and membrane potential but impairs cellular bioenergetics. (A)** Experimental design. BEAS2B cells were treated with several concentrations of GOx for 1 h and various analysis were preformed immediately after treatment or 24 h later. **(B)** GOx-treatment induces time dependent oxidative stress in BEAS 2B measured with CM-$H_2$DCFDA fluorescent dye. (**C**) Integrity of the nuclear DNA. (**D**) Permeability of the plasma membrane measured by LDH release. **(E)** Mitochondrial volume measured by analysis of specific activity of citrate synthase. **(F)** Mitochondrial membrane potential. **(G)** Changes in mitochondrial respiration. (**H**) Changes in glycolytic function. (**I**) Level of mitochondrial superoxide was visualized using MitoSOX staining. (**J**) Effect of GOx-treatment on IL-6 expression in control and mtDNA depleted (rho0) A549 cells. Lack of mtDNA was confirmed by PCR using mitochondrial and nuclear DNA specific primers. NC, negative control (lack of DNA template) of PCR reaction. Data represent average±SEM of n=5 biological replicates. Representative images of n=3 independent experiments are shown. * $p<0.05$, ** $p<0.01$ *vs.* control.

**A.**

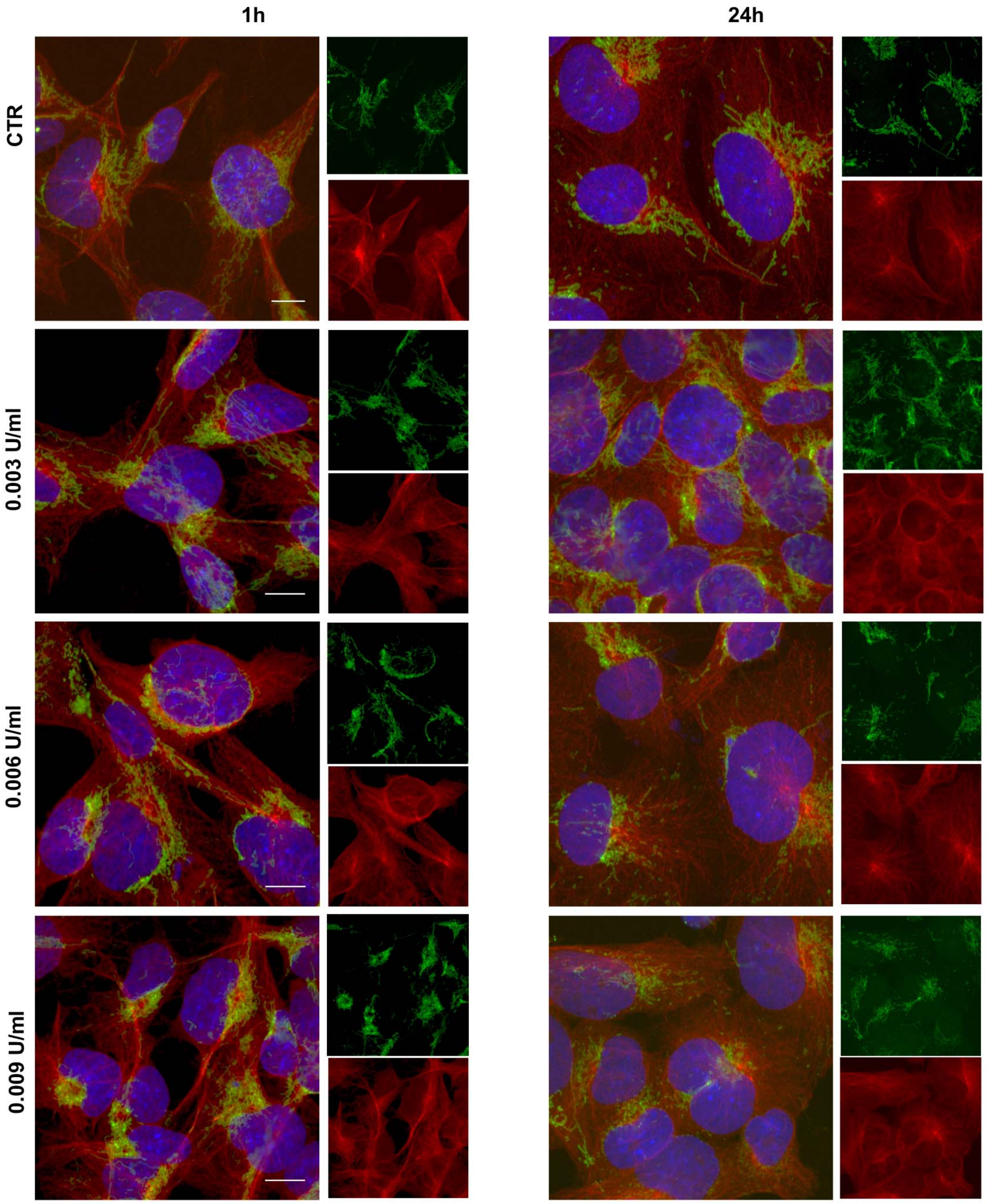

**B.**

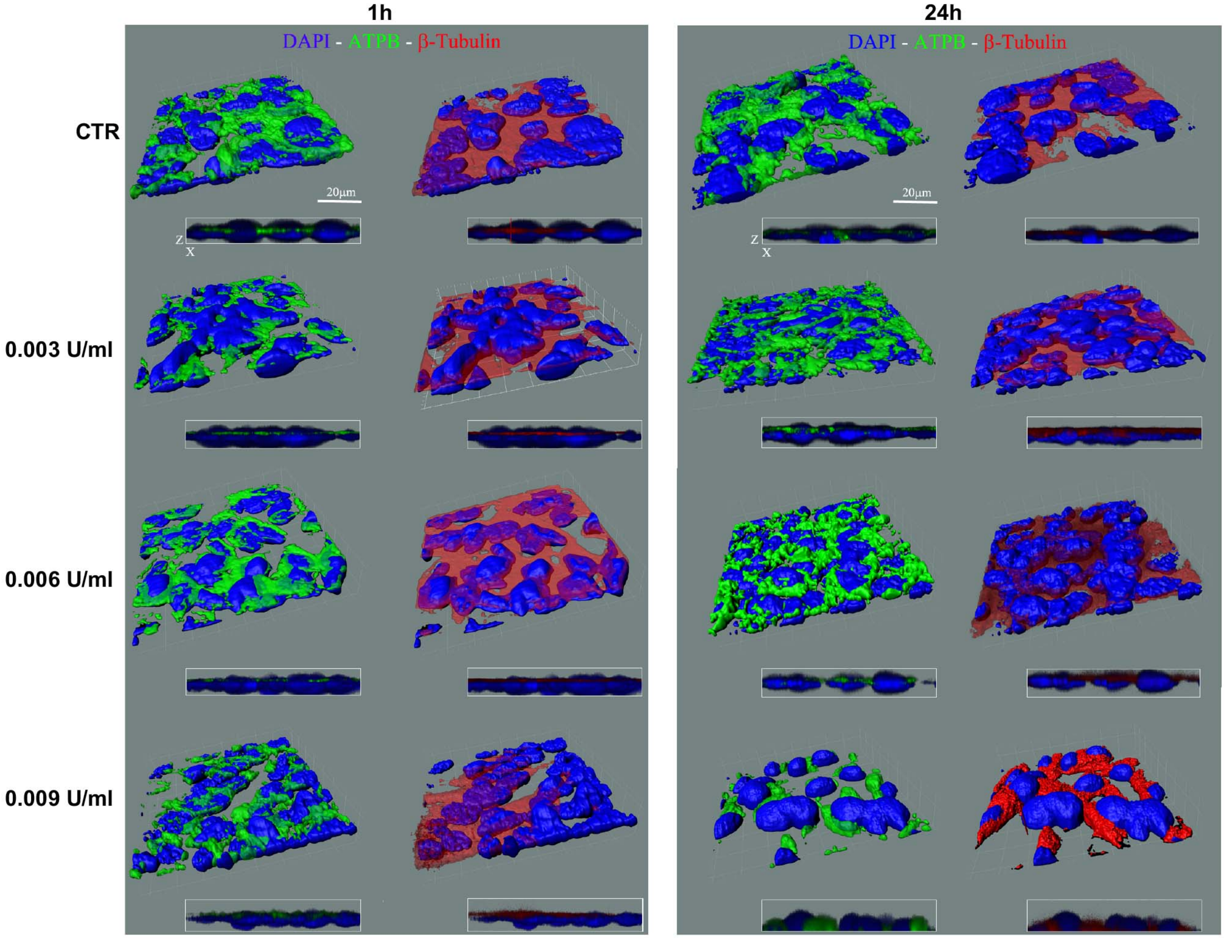


**C.**

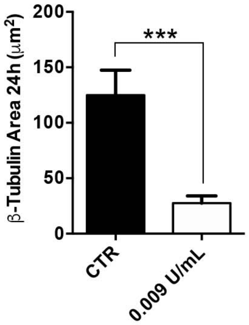


**Fig. S2**. **Non-cytotoxic level of oxidative stress induces detectable changes in cellular morphology. (A)** Effect of oxidative stress on changes in cellular morphology visualized by staining nucleus (DAPI, blue), mitochondrial network (ATPB, green) and cytoskeleton (β-tubulin, red;). **(B)** 3-D reconstruction of the mitochondrial network and cellular cytoskeleton (staining ATPB, green; nucleus, DAPI blue, cytoskeleton b-tubulin red). (**C**) Quantification of

tubulin-specific area in control and GOx-treated BEAS 2B cells at 24h. Representative images of n=3 independent experiments are shown.

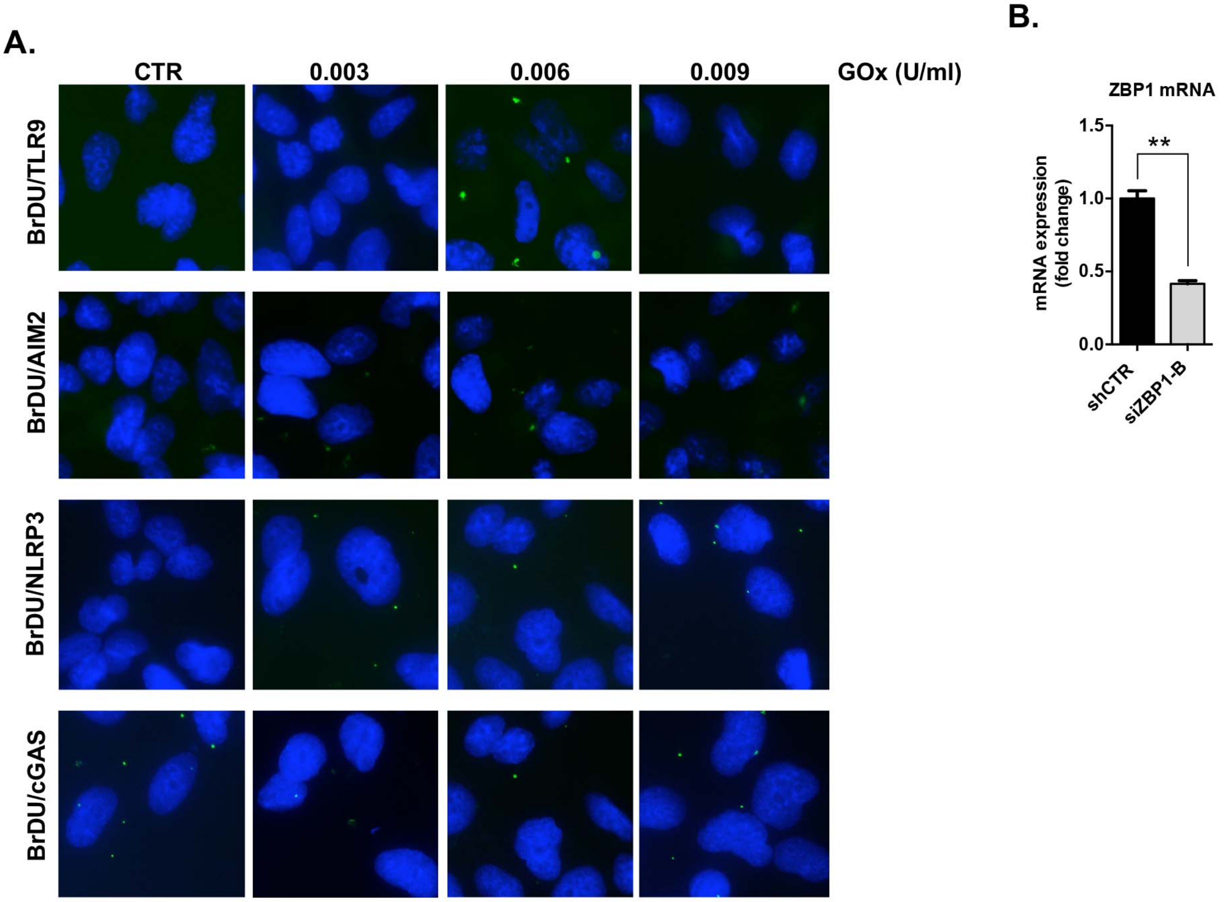


**Fig. S3. (A)** Damaged mtDNA does not interact with TLR9, AIM2, NLRP3 or cGAS in GOx-treated BEAS2B cells. Representative images of n=3 independent determinations are shown. (**B**) ZBP1 mRNA level in BEAS 2B cells transfected with siZBP1-B at 48 h post-transfection.

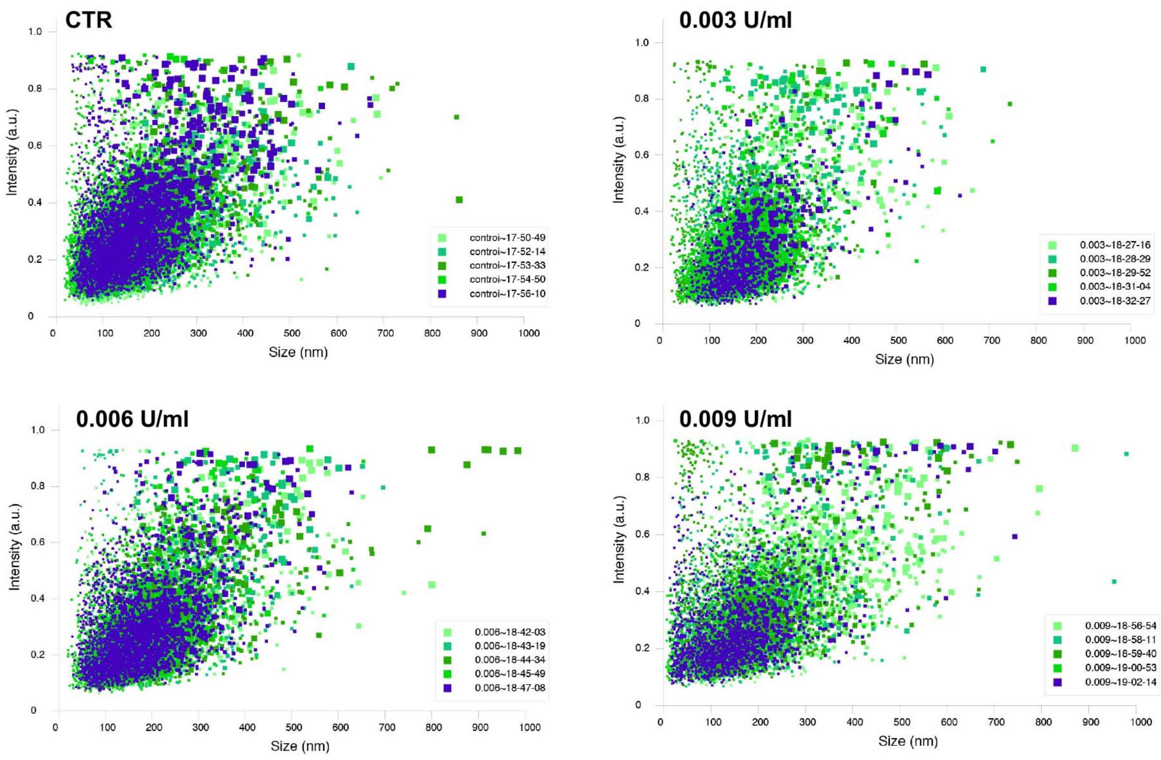


**Fig. S4. Analysis of nanoparticles at 24 h in control and GOx-treated BEAS 2B cells.**

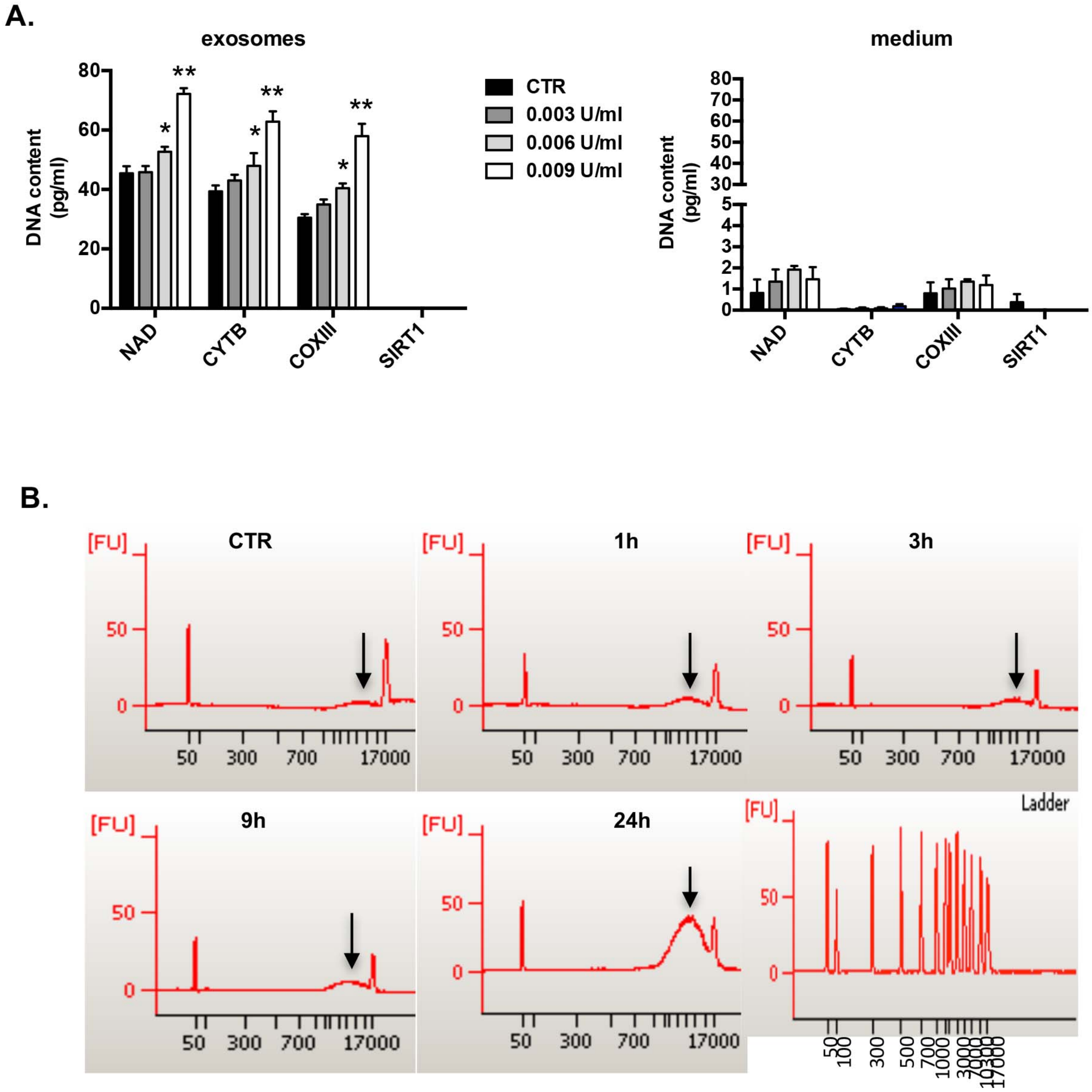


**Fig. S5. Large mitochondrial DNA fragments are released from oxidatively stressed BEAS2B cells.** (**A**) The amount of the mtDNA and nuclear DNA in exosomal fraction and remaining media at 24 h post GOx treatment. Data represent average±SEM of n=5 biological replicates. * p<0.05, ** p<0.01 *vs.* control. (**B**) Analysis of the mtDNA fragments size in exosomal fraction of BEAS2B cells treated with 0.006 U/ml of GOx at 0, 1, 3, 9 and 24 h post treatment. Sixth panel including DNA marker, range 50 bp – 17,000bp, as indicated below.

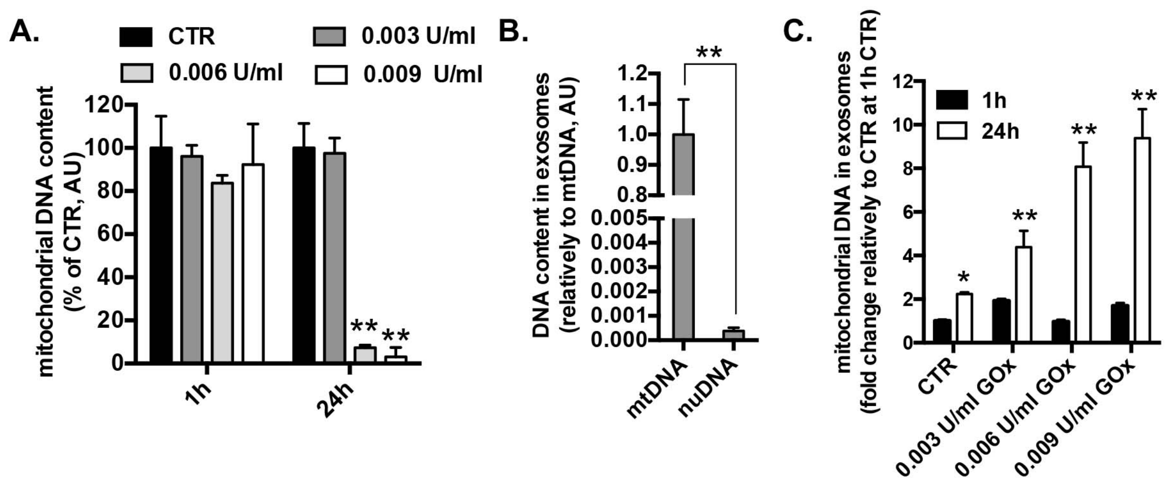


**Fig. S6. Mitochondrial DNA release from oxidatively stressed A549 cells**. (**A**) MtDNA content at 1 and 24 h in control and GOx-treated A549 cells. (**B**) Comparison of the amount of the mitochondrial and nuclear DNA in exosomes isolated from medium of A549 cells at 24 h post 0.006 U/ml GOx treatment. (**C**) Relative amount of the mitochondrial DNA in exosomal fraction of A549 cells treated with GOx at 1 h and 24 h. Data represent average±SEM of n=5 (n=3 for B) biological replicates. * $p<0.05$, ** $p<0.01$ *vs.* control.

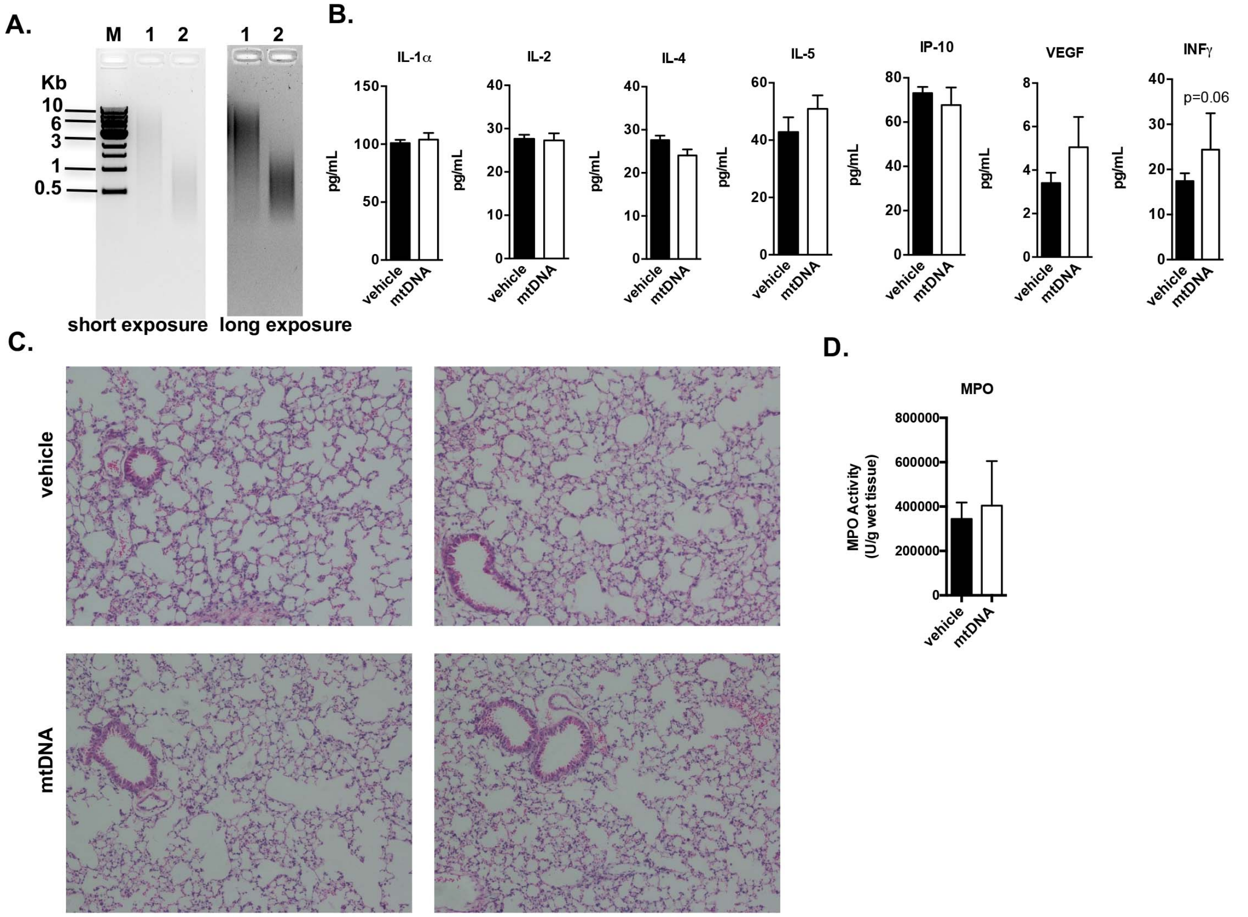


**Fig. S7. Injection of the mtDNA does not induce lung injury *in vivo*. (A)** Agarose gel analysis of the mtDNA isolated from liver (1) and mtDNA fragments generated by sonication (2). M, marker DNA ladder. (**B**) Expression of cytokines/chemokines that did not change by the mtDNA injection. **(C)** Histology of lung tissue and **(D)** MPO activity in lung homogenates of mice treated with vehicle or isolated mtDNA. 6-8 animals were used for each experimental end-point. Data represent average±SEM.

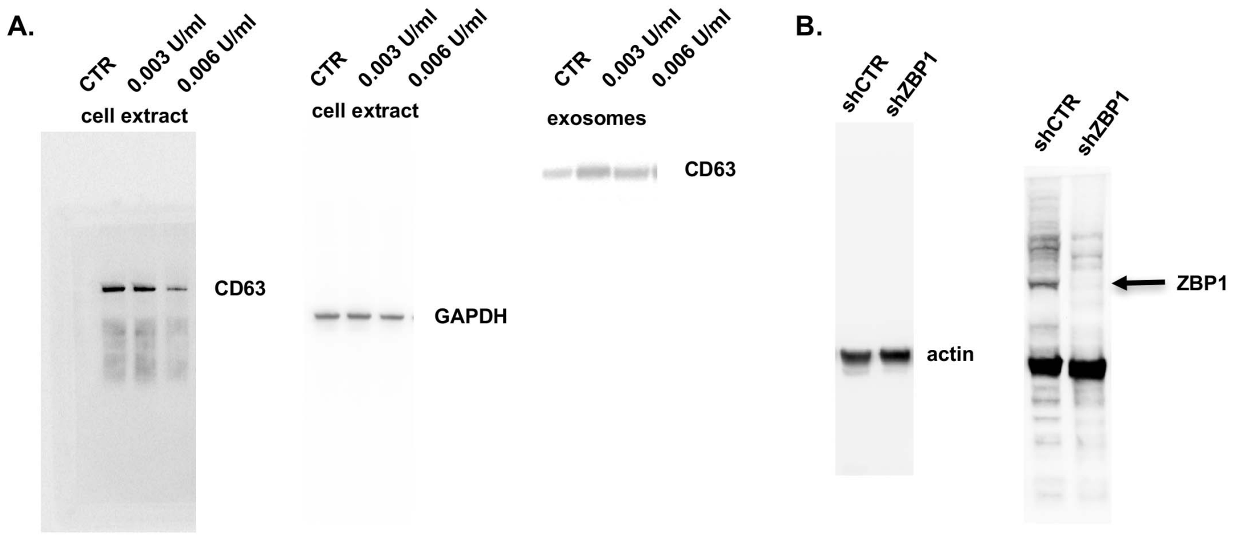


**Fig. S8. Entire images of Western blots used in this study.** (**A**) Uncropped Western blot images used in Fig. 4C. (**B**) Uncropped Western blot images used in Fig. 3E.
